# Associations of Markers of Inflammatory Status and Adiposity with Bone Phenotype at Age 60–64 Years: Findings from the MRC National Survey of Health and Development

**DOI:** 10.1007/s00223-025-01380-y

**Published:** 2025-05-06

**Authors:** Ruth Durdin, Camille Pearse, Diana Kuh, Rachel Cooper, Elaine M. Dennison, Cyrus Cooper, Kate A. Ward

**Affiliations:** 1 MRC Lifecourse Epidemiology Centre, University of Southampton, Southampton General Hospital, Southampton, UK; 2 National Institute for Health Research Southampton Biomedical Research Centre, University of Southampton and University Hospital Southampton NHS Foundation Trust, Southampton, UK; 3 MRC Unit for Lifelong Health and Ageing, University College London, London, UK; 4 AGE Research Group, Translational and Clinical Research Institute, Newcastle University, Newcastle Upon Tyne, UK; 5 NIHR Newcastle Biomedical Research Centre, Newcastle Upon Tyne Hospitals NHS Foundation Trust, Tyne and Wear NHS Foundation Trust and Newcastle University, Cumbria, Newcastle Upon Tyne, Northumberland UK; 6 Institute of Musculoskeletal Science, Nuffield Department of Orthopaedics, Rheumatology and Musculoskeletal Science, University of Oxford, Oxford, UK

**Keywords:** Dual energy X-ray absorptiometry, Peripheral quantitative computed tomography, Bone mineral density, Inflammation, Body composition, Ageing

## Abstract

This study investigated associations between markers of inflammatory status and adiposity (interleukin-6 [IL-6], adiponectin and leptin) and measures of bone phenotype and fractures. The Medical Research Council (MRC) National Survey of Health and Development (NSHD) is a British birth cohort study. Participants (born during the same week in 1946) with complete data on DXA and pQCT parameters, markers of inflammatory status and adiposity, and potential confounders (498 men and 474 women) were included in cross-sectional analyses. At age 60–64 years, bone phenotype was assessed by DXA and pQCT. Fractures were self-reported at ages 60–64 and 68–70 years. Multiple linear regression was used to determine associations of IL-6, adiponectin and leptin with bone phenotype (adjusted for fat and lean mass and lifestyle confounders). Standard deviation (SD) differences in outcomes per SD increases in exposures were estimated. Higher IL-6 levels were associated with lower total volumetric bone mineral density (vBMD) (− 0.10[− 0.19, 0.00]) in men, and higher areal BMD (aBMD) at the spine (0.12[0.03, 0.22]) and whole body (0.11[0.01, 0.20]) in women. Higher levels of adiponectin were associated with lower aBMD and trabecular vBMD. In women, higher leptin levels were associated with higher cortical vBMD (0.11[0.02, 0.20]). Higher adiponectin was associated with moderately increased odds of having a fragility fracture during adulthood in women (OR 1.16 [95% CI 0.94, 1.43, *p* = 0.18]). Our results highlight non-mechanical associations between markers of inflammatory status and adiposity with BMD and, in women, fractures. Ensuring inflammaging is minimised may be important in healthy bone ageing.

## Introduction

Osteoporosis, which is characterised by the loss of bone mass and deterioration of bone microarchitecture [1], is of major concern given the considerable morbidity, mortality and economic costs associated with fragility fractures [2]. To reduce these burdens at the individual and healthcare level, it is important to further investigate the determinants of poorer bone health in older age.

A range of risk factors including genetic factors, co-morbidities and lifestyle factors, such as smoking status and alcohol intake, contribute to the loss of bone mass with age [3]. Inflammation, a chronic low-grade level of which is associated with ageing, is also suggested as a risk factor for several chronic conditions [4]. Increased adiposity, in particular visceral, is associated with an inflammatory state via the release of mediators such as interleukin-6 (IL-6) [5]. Adipokines, leptin and adiponectin, are also released from adipose tissue and are positively and negatively associated with fat mass, respectively; in addition to their roles as hormones, they also have pro- and anti-inflammatory actions [6]. Links between inflammation and bone cell metabolism have been described, suggesting that the immune system may play a role in the development of osteoporosis [7]. Higher levels of inflammatory markers have also been associated with an increased risk of fracture [8–11].

Previously reported relationships between inflammatory markers and dual energy X-ray absorptiometry (DXA)-measured bone mineral density (BMD) have been varied. IL-6 has been inversely associated with BMD [12] and, in the Health, Aging and Body Composition (Health ABC) study, men and women with the highest levels of IL-6 were shown to have up to 39% higher risk of fracture compared to individuals with lower levels [11]. Leptin acts indirectly on bone via the central nervous system, as well as directly acting on bone cells [13]. Previously, clinical studies have demonstrated positive associations between leptin and BMD [14, 15]; however, negative associations have also been reported [16]. Finally, although the direct and indirect action of adiponectin on bone cells also differs [17, 18], various clinical studies have reported associations between higher adiponectin and lower BMD [19, 20]. Men with higher adiponectin levels (tertile 3: 11.0–53.0 ug/ml) were shown to have a 94% higher risk of fracture compared to individuals with lower levels (tertile 1: 1.0–6.0 ug/ml)) [21].

Relatively few studies have investigated the links between adipokines or inflammatory markers and other aspects of bone phenotype in addition to DXA-measured BMD, such as the relationships with individual bone compartments (cortical and trabecular bone). We selected the markers IL-6, adiponectin, and leptin given their relationships with inflammation, release from adipose tissue, and contrasting positive and negative relationships with bone metabolism. There may be associations between these markers and individual bone compartments which are not identified by DXA. Previous studies have included the demonstration of poorer trabecular bone microarchitecture in older men with high C-reactive protein (CRP) levels [22], as well as negative associations between leptin and cortical bone size in younger men [23]. In postmenopausal women, adiponectin and leptin were negatively associated with total volumetric BMD (vBMD) and bone cross-sectional area, respectively [24].

The aim of this study was to investigate the relationships between IL-6, adiponectin and leptin and DXA and peripheral quantitative computed tomography (pQCT) measures of bone density, size and strength, as well as fragility fractures, in men and women.

## Materials and Methods

### Study Population

The Medical Research Council (MRC) National Survey of Health and Development (NSHD) is a prospective birth cohort study; at baseline, the study consisted of 2815 males and 2547 females and all participants were born in England, Scotland or Wales during the same week in March 1946 [25]. The participants included in these analyses were members of the cohort who were still alive and living in Britain and attended the study visit when they were aged 60–64 years. As previously described, study members were eligible for inclusion in the study visit at age 60–64 years if they were living in England, Scotland or, Wales, had not previously withdrawn, died or were not untraced in the study [25]. At this study visit, participants were invited for assessment at one of six clinical research facilities (CRF) or, if unable or unwilling to attend, participants were offered a home visit. Ethics committee approval was given for the follow-up at age 60–64 years, during which musculoskeletal assessment took place, by the Central Manchester Research Ethics Committee. Ethics approval for the 2014–2016 follow-up was given by the NRES Committee London–Queen Square. Participants gave written informed consent.

### Musculoskeletal assessment

During the CRF visit, participants had DXA scans using QDR 4500 Discovery machines (Hologic Inc, Bedford, MA). Measurements included bone (lumbar spine [L1-L4], total hip and whole body areal BMD [aBMD] g/cm^2^) and body composition (lean and fat mass kg). Using an XCT 2000 scanner (Stratec Medizintechnik, Pforzheim, Germany), pQCT scans of the non-dominant radius were also obtained. The pQCT outcomes were trabecular vBMD (mg/cm^3^) and total vBMD (mg/cm^3^) at the 4% site, and cortical vBMD (mg/cm^3^), cortical cross-sectional area (CSA) (mm^2^), total CSA (mm^2^) and medullary CSA (mm^2^) at the 50% site. In addition, polar stress strain index (SSI), which provides an estimate of bone strength, was calculated at the 50% site. Details of scan acquisition and cross-calibration have been previously described [26].

### Clinical Measurements and Questionnaires

Details of the measurements and samples collected during the clinical visit have been described previously [25]. Height (measured using a stadiometer [CMS Weighing equipment Ltd, London, UK]) and weight (measured using Tanita Solar weighing scales [Tanita UK, Ltd, Uxbridge, UK]) were measured according to standard protocols. An overnight fasting blood sample was taken either during the clinic visit or at home. Blood samples were stored at − 80 °C [25]. CRP was measured, at MRC Human Nutrition Research, Cambridge, UK, using an immunoturbidimetric assay (Siemens Dimension Xpand) using the manufacturer’s reagents. Other biomarkers were undertaken by British Heart Foundation Research Centre, Glasgow, UK; IL-6 was measured using high sensitivity enzyme-linked immunosorbent assay (ELISA) (R&D Systems, Biotechne, Oxon, UK), adiponectin was measured using ELISA (R&D Systems, Bio Techne, Oxon, UK) and leptin was measured using an in-house method measured against commercially available kits. Intra-assay co-efficients of variation are provided in Murray et al. [27].

Fractures were self-reported in questionnaires collected during the 2006–2010 and 2014–2016 study visits. Fracture data (including the site and mechanism) were collected via pre-assessment and postal questionnaires, during the 2006–2010 and 2014–2016 study visits, respectively. During this study, fragility fractures were defined by site (clavicle, rib, sternum, humerus proximal, distal radius, vertebra, pelvis or proximal femur) or mechanism (the result of a fall from standing height, below standing height or no trauma) based on self-report of fractures since age 25.

Data on smoking status (smoking history–current, ex or never–up to age 64 years), physical activity (leisure time physical activity in the last 4 weeks at age 60–64 years) [28], social class (based on occupation of the head of household at age 53) [29] and, in women, ever-use of hormone replacement therapy (HRT) [30] was also collected.

### Statistics

The study population for these analyses included individuals with complete data available for the markers of inflammatory status and adiposity (IL-6, adiponectin and leptin), the DXA and pQCT parameters and the lifestyle- and body composition-confounders (smoking status, physical activity, social class, HRT use in women, fat for lean mass residual and leptin residual) (maximum *n* = 972 [given that data for the secondary fracture analysis were from the questionnaires, individuals without questionnaire data were therefore excluded from the fracture analysis so *n* = 950 for fracture analysis]). Descriptive statistics are given as mean (standard deviation [SD]) for normally distributed variables or median (interquartile range [IQR]) for skewed variables. Leptin and IL-6 were highly skewed and were log transformed prior to analysis.

Multiple linear regression models were used to test the associations between markers of inflammatory status and adiposity and DXA- and pQCT-measured bone phenotype, with standardised outcomes and predictors produced for men and women separately. Models containing log(IL-6) and adiponectin were adjusted for fat mass residuals. Fat mass residuals were generated by regressing fat mass on lean mass and height. This enabled the effect of fat mass, which remained after adjustment for lean mass and height, to be accounted for [31]. Leptin was highly correlated with the fat mass residual (men: *r* = 0.61, women: *r* = 0.61, both *p* < 0.01); therefore, leptin residuals were generated instead by regressing log(leptin) on fat mass and height. Models with leptin residuals as the exposure were also adjusted for lean mass given that it was not a component of the leptin residual. All leptin associations explored in this study are based on these leptin residuals. For all models, beta is interpreted as SD change in the bone outcome per SD increase in inflammatory marker; *p* < 0.05 was regarded as statistically significant. All models were also adjusted for smoking status, physical activity, social class and, in women, ever-use of HRT.

Logistic regression, using the same lean and fat mass adjustments as described for the multiple linear regression models, was used to determine odds ratios for the association between each inflammatory marker and self-report of at least one fragility fracture at any timepoint during adulthood, reported during the 2006–2010 and 2014–2016 study visits.

Analyses were conducted using Stata Version 17.0 (StataCorp, College Station, TX, USA). Figures were created using GraphPad Prism.

## Results

### Descriptive Characteristics

Descriptive characteristics of the 972 participants (498 men and 474 women) included in these analyses are given in Table 1. The mean (SD) age of men and women in this study was 62.9 (1.2) and 63.0 (1.1) years, respectively. BMI was similar in men (mean: 27.8 kg/m^2^) and women (mean: 27.6 kg/m^2^). Adiponectin and leptin levels were higher in women, whereas IL-6 levels were higher in men.

**Table 1:** Descriptive characteristics for men and women separately

Background and markers of inflammatory status and adiposity		
	Men (*n* = 498)	Women (*n* = 474)
	Mean (SD)	Mean (SD)
Age (years)	62.9 (1.2)	63.0 (1.1)
Height (m)	1.8 (0.1)	1.6 (0.1)
Weight (kg)	85.2 (13.0)	72.7 (13.6)
BMI (kg/m^2^)	27.8 (4.0)	27.6 (5.0)
Adiponectin (ug/ml)	10.7 (7.2)	18.7 (9.9)

	Median (IQR)	Median (IQR)
Leptin (ng/ml)	7.0 (4.1, 10.7)	19.0 (10.8, 31.8)
IL-6 (pg/ml)	2.0 (1.3, 3.1)	1.9 (1.3, 2.9)

Bone parameters		
	Mean (SD)	Mean (SD)
DXA		
Spine aBMD (g/cm^2^)	1.04 (0.18)	0.94 (0.16)
Total hip aBMD (g/cm^2^)	1.00 (0.14)	0.87 (0.12)
Whole Body aBMD (g/cm^2^)	1.14 (0.1)	1.04 (0.09)
pQCT		
4% site		
Trabecular vBMD (mg/cm^3^)	205.02 (42.59)	172.9 (40.43)
Total vBMD (mg/cm^3^)	390.88 (66.29)	332.12 (68.91)
50% site		
Cortical vBMD (mg/cm^3^)	1157.81 (35.13)	1149.03 (37.26)
Cortical CSA (mm^2^)	111.11 (14.62)	77.68 (10.71)
Total CSA (mm^2^)	154.18 (22.14)	112.86 (15.98)
Medullary CSA (mm^2^)	43.07 (14.72)	35.18 (12.12)
Polar SSI (mm^3^)	346.41 (69.75)	212.98 (43.45)

Fragility fractures^a^	*N* (%)	*N* (%)
Yes	73 (15.0)	118 (25.4)
No	413 (85.0)	346 (74.6)

^a^At least one fragility fracture self-reported during adulthood between the 2006–2010 and 2014–2016 study visits (individuals without questionnaire data excluded); 486 men and 464 women had fracture data

*aBMD* areal bone mineral density, *BMI* body mass index, *CSA* cross-sectional area, *DXA* dual energy X-ray absorptiometry, *IL-6* interleukin-6, *IQR* interquartile range, *pQCT* peripheral quantitative computed tomography, *SD* standard deviation, *SSI* stress strain index, *vBMD* volumetric bone mineral density

### Associations of IL-6, Adiponectin and Leptin with DXA- and pQCT-Measured Bone Parameters

*IL-6* (Fig. 1a).

**Fig. 1:**
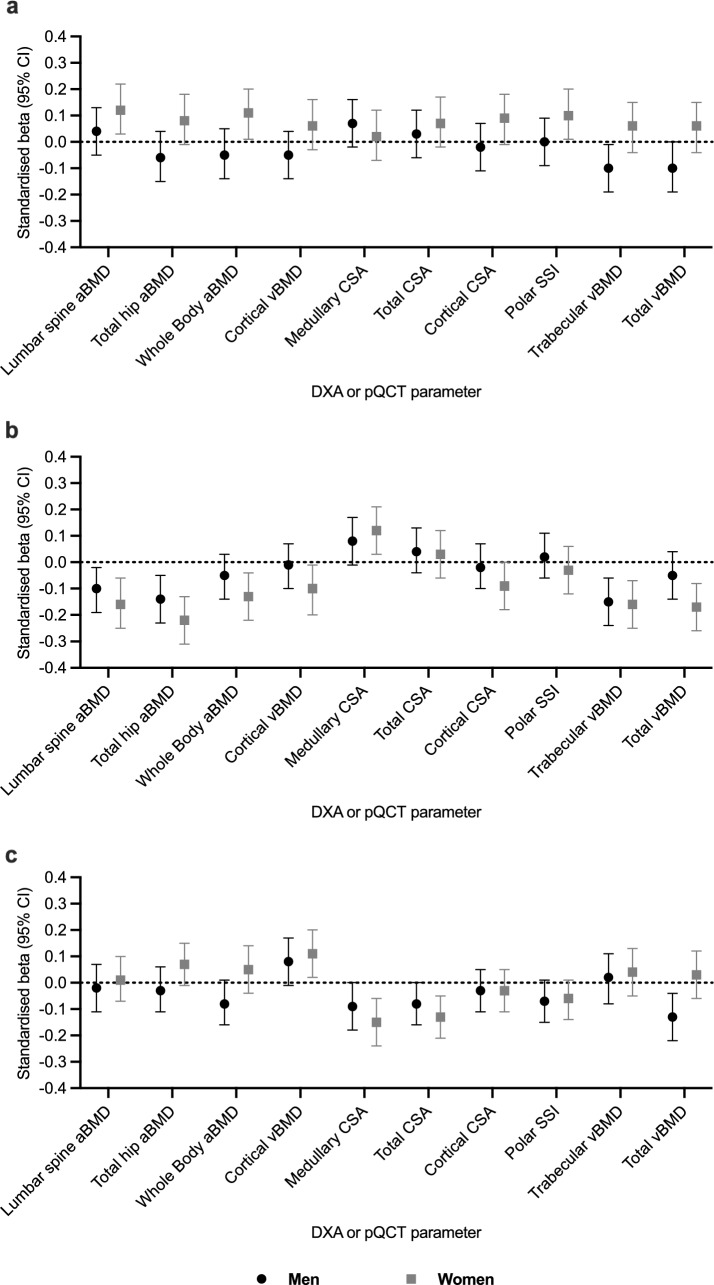
Associations between **a** IL-6, **b** adiponectin and **c** leptin with DXA and pQCT parameters in men and women separately (**a** and **b**) adjusted for fat mass residual, smoking status, physical activity, social class and (in women only) HRT ever used; (**c**) adjusted for lean mass, smoking status, physical activity, social class and (in women only) HRT ever used. Beta is interpreted as SD change in the bone outcome per SD increase in inflammatory marker. *aBMD* areal bone mineral density, *CI* confidence interval, *CSA* cross-sectional area, *DXA* dual energy X-ray absorptiometry, *IL-6* interleukin-6, *pQCT* peripheral quantitative computed tomography, *SSI* stress strain index, *vBMD* volumetric bone mineral density

In men, higher IL-6 levels were associated with lower trabecular vBMD (− 0.10 [− 0.19, − 0.01]) and total vBMD (− 0.10 [− 0.19, 0.00]). In women, higher IL-6 levels were associated with higher aBMD at the spine (0.12 [0.03, 0.22]) and whole body (0.11 [0.01, 0.20), and with greater polar SSI (0.10 [0.01, 0.20]).

*Adiponectin* (Fig. 1b).

In men, higher adiponectin levels were associated with lower aBMD at the spine (− 0.10 [− 0.19, − 0.02]) and total hip (− 0.14 [− 0.23, − 0.05]). Higher levels of adiponectin were also associated with lower trabecular vBMD (− 0.15 [− 0.24, − 0.06]). In women, higher adiponectin levels were associated with lower aBMD at all sites (spine: − 0.16 [− 0.25, − 0.06]; total hip: − 0.22 [− 0.31, − 0.13]; whole body: − 0.13 [− 0.22, − 0.04) and with lower trabecular vBMD (− 0.16 [− 0.25, − 0.07]) and total vBMD (− 0.17 [− 0.26, − 0.08]), and greater medullary CSA (0.12 [0.03, 0.21]).

*Leptin* (Fig. 1c).

In men, higher leptin levels were associated with lower total vBMD (− 0.13 [− 0.22, − 0.04]) and, in women, with higher cortical vBMD (0.11 [0.02, 0.20]), as well as with lower medullary CSA (− 0.15 [− 0.24, − 0.06]) and lower total CSA (− 0.13 [− 0.21, − 0.05]). There was a relationship between leptin and total hip aBMD (0.07 [− 0.01, 0.15]) in women, which did not reach the 5% significance level (*p* = 0.07).

### Associations of IL-6, Adiponectin and Leptin with Fragility Fracture

**Fig. 2:**
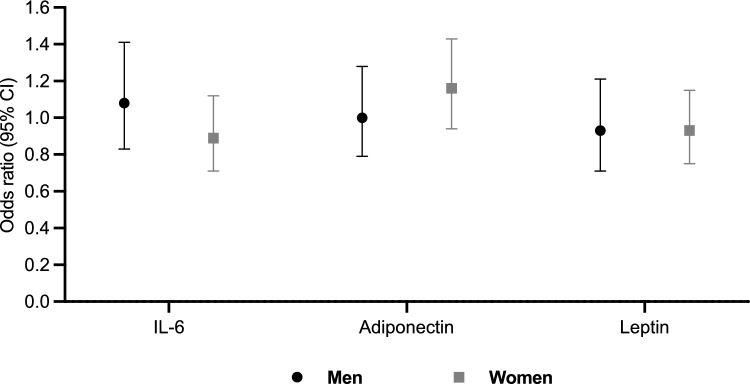
Associations between IL-6, adiponectin and leptin and fragility fracture as OR (95% CI) for men and women separately. Models containing IL-6 and adiponectin adjusted for fat mass residuals; model containing the leptin residual adjusted for lean mass. All models adjusted for smoking status, physical activity, social class and (in women only) HRT ever used. *CI* confidence interval, *IL-6* interleukin-6, *OR* odds ratio

Table 1 shows the prevalence of fragility fractures in men and women separately. In women, although the association did not reach statistical significance, higher adiponectin was associated with the odds of reporting a prevalent fragility fracture during adulthood (Odds ratio [OR] 1.16 [0.94, 1.43] *p* = 0.18) (Fig. 2).

## Discussion

In this study, we have investigated associations between markers of inflammatory status and adiposity and bone phenotype in men and women. Overall, these data suggest modest associations between some markers of inflammatory status and adiposity, which together with an increasing number of risk factors, may influence healthy bone ageing. The use of both pQCT and DXA data helps to elucidate these relationships beyond those with DXA aBMD and, therefore, contributes to our understanding of the specific relationships with different aspects of bone phenotype. Higher adiponectin was also associated with the odds of having a fragility fracture during adulthood in women; however, this association did not reach statistical significance.

Higher IL-6 levels were associated with higher aBMD at the spine and whole body in women; in men, higher IL-6 levels were associated with lower trabecular and total vBMD. In the Hertfordshire Cohort Study, there were no associations between IL-6 and lumbar spine or femoral neck aBMD in cross-sectional analyses; these results were adjusted for gender and are consistent with the results which we found in men in NSHD [32]. Similarly, results from the Framingham Osteoporosis Study, showing a lack of associations between IL-6 and aBMD at the hip and spine, are also in agreement with our findings. The relationships which we found between higher IL-6 and higher aBMD at the spine and whole body in women are not consistent with results in the literature which report a negative, or lack of, association between IL-6 and aBMD [33, 34]. Interestingly, in the Framingham Osteoporosis Study, a relationship between CRP (an acute phase inflammatory marker downstream of IL-6) and higher femoral neck aBMD was observed in postmenopausal women using menopausal hormone therapy [35]. It is possible that the associations were due to another non-mechanical factor in the relationship between fat mass and bone or differences in age between studies. One of the strengths of NSHD is that all participants are born within a week of one another, limiting confounding by age.

We observed that higher adiponectin was related to lower aBMD in men and women which is consistent with findings from the Hertfordshire Cohort Study: at the lumbar spine, an SD increase in adiponectin was associated with 0.11 [− 0.21, − 0.01] lower aBMD in fully adjusted models, which included adjustment for gender, age, height, weight adjusted for height residuals, smoking, alcohol, diet, physical activity and hormone replacement therapy [32]. After full adjustment, we observed a similar effect size at the spine in men (− 0.10 SD [− 0.19, − 0.02]) and women (− 0.16 SD [− 0.25, − 0.06]). Associations between adiponectin and pQCT-measured vBMD were also previously reported in the InChianti study [20]. Similar to our results, relationships were observed between adiponectin and cortical, trabecular and total vBMD in women; however, in contrast, there were no associations in men, and there was a significant interaction between gender and adiponectin in the relationship with cortical and trabecular vBMD. Finally, in another study in postmenopausal women (mean age: 62.3 years), adiponectin was negatively associated with total vBMD of the distal tibia [24]. This is consistent with the relationship we observed between adiponectin and total vBMD at the 4% radius in women in NSHD.

The direction of association between leptin and aBMD is consistent with results from the Rancho Bernardo study, in which higher leptin was related to higher aBMD in women only [15]. In addition, in the Rancho Bernardo study there was also an association at the lumbar spine. In the Hertfordshire Cohort Study, there were positive associations between leptin and femoral neck aBMD with adjustment for gender, but these were attenuated after adjustment for lifestyle factors [32]. In our study, a relationship between higher leptin levels and lower total vBMD was observed in men. Similar to our study, in young men (mean age: 18.9 years) there were also no associations between leptin and cortical vBMD or trabecular vBMD of the radius in fully adjusted models; however, in contrast to our results this study reported a negative association between leptin and cortical CSA [23]. In postmenopausal women, leptin concentration was negatively associated with total CSA of the tibia diaphysis [24]. Consistent with this, in our study, we found a relationship between higher leptin levels and lower total CSA at the 50% radius, which may help to demonstrate the contrasting direct and indirect effects of leptin on bone.

During our study, we found small effect sizes in the relationships between IL-6, leptin, and adiponectin, and bone phenotype. Clinically, DXA-measured BMD is a known predictor of fracture risk and, at age 65, per SD decrease in hip BMD, the risk of osteoporotic fracture increases by 1.41 (risk ratio: 1.41 [95% CI 1.33–1.51]) in men and by 1.38 in women (risk ratio: 1.38 [95% CI 1.28–1.48]) [36].

Associations of the markers of inflammatory status and adiposity with DXA and pQCT parameters did not translate into associations with prevalent fragility fractures. Similarly, in the Cardiovascular Health Study there was no association between IL-6 and a composite of incident fractures (including hip, pelvis, humerus or proximal forearm) in men and women [10]. In the Health ABC study, inflammatory markers were associated with an increased risk of fracture; however, the association between IL-6 and incident fracture risk was not statistically significant after adjustment for confounders [11]. In contrast to our results, in the Study of Osteoporotic Fractures, women in the highest IL-6 quartile had a greater risk of fracture compared to women in the lower IL-6 quartiles [9]. For adiponectin, negative associations with BMD, but not with fracture risk, have been shown [37]. Interestingly, in the Health ABC study, higher adiponectin levels in men, but not women, were associated with an increased risk of fracture after adjustment for confounders; however, consistent with our results, leptin was not associated with fracture risk in men or women [21]. Sex differences in the association between adiponectin and vertebral fractures were reported in the Rancho Bernardo Study, with higher adiponectin levels associated with an increased risk of vertebral fractures in men but not women [38]. Previously published results concerning the relationship between these markers of adiposity and inflammation with fracture are therefore conflicting. It is also possible that some of the differences between these studies could be due to differences in the type of fracture, such as traumatic or non-traumatic, or fracture sites, included in the studies.

In addition to clinical studies, preclinical studies have shown direct links between IL-6, adiponectin, and leptin and bone metabolism. IL-6 is known to induce osteoclast formation in vitro [39]. In addition, there are also links between IL-6 and osteoblasts, and IL-6 knockout mice showed enhanced bone formation following stress fractures [40]. Adiponectin has been shown to inhibit osteoclast formation in vitro [41] and adiponectin-deficient mice were shown to have reduced bone mass [42]. It is known that the action of leptin on bone varies depending on its direct and indirect action, which accounts for the fact that contrasting effects have been observed [13]. In vitro, leptin has been shown to inhibit osteoclast generation [43] and leptin-deficient mice were shown to have decreased bone growth and lower osteoblast-lined bone perimeter, both of which increased when leptin was administered [44].

Hormonal mechanisms may explain the differences in relationships between markers of inflammation and bone between men and women. For example, it is well-established that estrogen plays a major role in bone metabolism and, as an inhibitor of bone resorption, the loss of estrogen helps to explain bone loss in postmenopausal women [45]. It is also possible that additional aspects of body composition contribute to differences in bone health between men and women. In addition to body composition, there are also changes in measures of muscle strength and function, such as grip strength, with age in later life, the change in which shows correlations with hip BMD [46]. Of note, android to gynoid fat mass ratio, a marker of adipose distribution, has been shown to have negative associations with DXA-measured BMD in women but not men [47].

Indeed, it has been suggested that the relationship between fat and bone mass shows differences between men and women [48]. This is in agreement with the differences in the relationships which we found and, in addition, we found higher adiponectin levels in women compared to men. In addition, it has been shown that different adipose tissue deposits have different relationships with markers of adiposity, for example, visceral adipose tissue has a greater association with adiponectin, while subcutaneous adipose tissue has a greater association with leptin [49]. These differences may play an important role in the differences in the relationship between adiposity, inflammation, and bone.

A limitation of this study was that our sample did not include all NSHD participants who were assessed at age 60–64 years (maximum population size at 60–64 years: men: 1067, women: 1161; analysis sample size: men: 498, women: 474) and this limited our statistical power, and likely introduced bias, to detect associations. A further limitation is that fragility fractures were defined based on self-report of a broken bone (at an osteoporotic site or by a low-trauma mechanism) since 25 years of age, and it is therefore possible that there could be recall bias. Although we adjusted for smoking status, physical activity, social class, and ever-use of HRT in women, it is also possible that additional confounders, such as medication use and diet, may also have had the potential to influence the results we observed. Regarding diet, calcium and vitamin D and protein intake are important aspects of diet, which are known to be related to bone health [1, 29]. In addition, following a Mediterranean diet, or a nutrient-rich diet, both of which include nutrients with anti-inflammatory action, is likely to have a protective effect on bone via the reduction of oxidative stress [29, 50]. It may also be possible that other medications, which act via inflammatory pathways, played a role in the relationships between inflammation and bone. Finally, it has been shown that different types of exercise have different effects on BMD [51]. It is therefore possible that the type of exercise which participants undertook, such as weight bearing or non-weight bearing, resistance exercise, played a role in these relationships.

Finally, with subjects having all been born in the same week of the same year, the results may not be generalisable to other cohorts, especially as the NSHD cohort was born in the early post-war period, with specific conditions which may not have been seen in later cohorts. However, it is also a strength of our study that all participants were born in the same week in March 1946, as there is confidence that the associations are not confounded by age.

The use of fracture data, in addition to clinical measurements, is also a strength of our study. A further strength was the use of fat mass residuals, which enabled adjustment for body composition without introducing multi-colinearity between highly correlated predictors. We also used a novel method, the calculation of leptin residuals, which enabled the effect of leptin which remains after adjustment for fat mass to be investigated. A final strength is that participants had both DXA and pQCT measures of bone phenotype. This enabled the comparison of DXA with pQCT, which is independent of body size and able to measure the density of the trabecular and cortical compartments separately. Our results showed that, in some instances, while there was no association between a marker and DXA aBMD, there were associations with pQCT parameters.

## Conclusions

This study contributes to our knowledge of the relationships between inflammatory status, adiposity and bone using both DXA and pQCT, as well as fractures, in the same study population. This helps to further our understanding of non-mechanical links between body composition and bone, which may influence healthy ageing, and highlights the additional information which different aspects of bone phenotype, obtained from DXA, pQCT and fracture data, were able to provide. Further research in this area is likely to improve our understanding of the potential mechanisms to be targeted for interventions to improve bone health in later life and ultimately reduce the burden of osteoporosis on individuals and society.

## Data Availability

Data used in this publication are available to bona fide researchers upon request to the NSHD Data Sharing Committee via a standard application procedure. Further details can be found at http://www.nshd.mrc.ac.uk/data (10.5522/NSHD/Q102; 10.5522/NSHD/S102A; 10.5522/NSHD/Q103). For Open Access, the author has applied a CC BY public copyright license to any Author Accepted Manuscript version arising from this submission.
